# Modular hybrid total hip arthroplasty. Experimental study in dogs

**DOI:** 10.1186/1751-0147-53-46

**Published:** 2011-07-07

**Authors:** Bruno W Minto, Cláudia Valéria S Brandão, Gilberto JC Pereira, Daniela Campagnol, Maria Jaqueline Mamprim, Carlos Roberto Padovani, José JT Ranzani

**Affiliations:** 1 Department of Veterinary Surgery and Anesthesiology, São Paulo State University (UNESP), School of Veterinary Medicine and Animal Science. Botucatu, PO Box 560, Rubião Júnior s/n Botucatu (SP) 18618-000, Brazil; 2 Department of Surgery and Orthopedics, São Paulo State University (UNESP), School of Medicine. Botucatu, PO Box 560, Rubião Júnior s/n Botucatu (SP) 18618-000, Brazil; 3 Department of Biostatistics, São Paulo State University (UNESP), Institute of Biosciences. Botucatu, PO Box 560, Rubião Júnior s/n Botucatu (SP) 18618-000, Brazil

**Keywords:** Dog, hip, total hip prosthesis, hybrid system, modular system

## Abstract

**Background:**

This prospective experimental study evaluated the surgical procedure and results of modular hybrid total hip arthroplasty in dogs.

**Methods:**

Ten skeletally mature healthy mongrel dogs with weights varying between 19 and 27 kg were used. Cemented modular femoral stems and uncemented porous-coated acetabular cups were employed. Clinical and radiographic evaluations were performed before surgery and at 30, 60, 90, 120, 180 and 360 days post-operation.

**Results:**

Excellent weight bearing was noticed in the operated limb in seven dogs. Dislocation followed by loosening of the prosthesis was noticed in two dogs, which were therefore properly treated with a femoral head osteotomy. Femoral fracture occurred in one dog, which was promptly treated with full implant removal and femoral osteosynthesis.

**Conclusions:**

The canine modular hybrid total hip arthroplasty provided excellent functionality of the operated limb.

## Background

Total hip arthroplasty (THA) is a common method of therapy for osteoarthrosis of the canine coxofemoral joint worldwide [1-4]. The main cause of coxofemoral ostearthrosis in dogs is hip dysplasia. This orthopedic disease is the most important cause of hindlimb lameness, pain and reduced quality of life in dogs [5,6]. Among the various treatments for hip dysplasia, THA stands out owing to its high success rate regarding alleviation of pain and restoration of hip function [7].

Only a few studies on THA have been accomplished in Brazil, especially because of difficulties involving importation of implants and lack of training by veterinary surgeons. Nevertheless, some studies describe the use of devices produced domestically in Brazil [8].

Several variations of canine THA techniques and implants are described in the veterinary literature [9]. Cemented, fixed-head THA implants were widely used in the 80's and early 90's [10], followed by modular prostheses [5,11] and more recently, cementless endoprostheses have been extensively investigated [12,13]. Canine cementless prostheses were developed by BioMedtrix and Kyon [4,14].

The clinical success following cemented or cementless THA in dogs is generally good as this procedure results in excellent hip joint function in 80% to 98% of the patients [9]. Complications after THA are infrequent but often difficult to manage [15]. Common complications include aseptic or septic implant loosening, dislocation, infection, and femoral fracture [6].

In dogs, THA results usually last for a lifetime [10] as the useful duration of the implants has been improved by the development of bone ingrowth prosthetic components [4,14].

Cementless fixation of the acetabular component has become increasingly popular because the long-term results of cemented THA have shown that late failure is due to loosening of the acetabular component [12]. Loosening of the prosthetic components is considered the most important cause of long-term THA failure in humans and dogs [12,16]. However, some difficulties and specific complications have been associated with the use of cementless stems [17]. Some studies in dogs used a hybrid combination of cementless acetabular cup and cemented femoral stem [12].

A hybrid combination is an important variation in cemented and uncemented THA in human orthopedics, but only few reports have been found in the veterinary literature. The present study aimed to evaluate the surgical procedure of THA using a modular hybrid total hip prosthesis developed in Brazil.

## Methods

THA in the left hindlimb was performed in 10 skeletally mature mongrel dogs. Body weights ranged between 19 and 27 kg among experimental animals and none exhibited hip and stifle injuries or any neurological disease.

All dogs remained with unilateral modular hybrid total hip prostheses during a period of 12 months. The animal protocol used in this investigation was approved by the Ethics Committee of the School of Veterinary Medicine and Animal Science, São Paulo State University -- UNESP Botucatu, SP, Brazil.

Modular hybrid total hip replacement prostheses composed of a cementless acetabular component and a cemented femoral stem (Cruz Alta Pro Hospitalar Ltda, Fernandópolis, Brazil) were used for the arthroplasties. The components were made of a cast cobalt-chromium alloy. The acetabular components were manufactured in diameters of 19, 21, 23, 25 and 27 mm and external surface was coated with a porous titanium fiber mesh. A modular cemented collarless femoral stem was made in three sizes (small, medium and large). The femoral heads were manufactured in standard and extended neck configurations (12 and 15 mm, respectively).

The perioperative approach and care followed the current standards and included checking for systemic infection by complete blood count, urinalysis and thorough clinical inspection. Cephalothin (22 mg/kg BW; Cefalin, EMS, São Paulo, Brazil) was intravenously administered 30 minutes before the skin incision; half of this dose was delivered intraoperatively at 90-minute intervals. Strict surgical aseptic techniques were used.

After premedication with intramuscular administration of morphine (0.3 mg/kg BW; Dimorf, Cristália, Itapira, Brazil) and acepromazine (0.05 mg/kg BW; Acepram, Univet, São Paulo, Brazil), anesthesia was induced with intravenous administration of propofol (4 mg/kg BW; Propovan, Cristália, Itapira, Brazil) and maintained with isoflurane in oxygen (Isothane; Baxter, Guayama, Puerto Rico). Caudal epidural anesthesia was induced with morphine (0.06 mg/kg BW; Dimorf, Cristália, Itapira, Brazil), and bupivacaine (0.05 mg/kg BW; Bupivacaina, Abbott, São Paulo, Brazil).

Using a craniolateral skin incision centered over the hip joint, the craniolateral aspect of the femoral neck was exposed by elevation of the vastus lateralis muscle. A T-shape capsulotomy was performed. After osteotomy and removal of the femoral head and neck, the proximal femur was retracted caudally. The acetabular bed was reamed with an appropriate size-specific reamer. The acetabular component was inserted manually and positioned with the help of an impactor. The acetabular component was then anchored in the acetabular bed with two or three stainless steel cortical screws, which were directed toward the cancellous bone of the dome of the acetabulum. An ultra-high molecular weight polyethylene insert was placed into the acetabular component.

The femoral canal was prepared by standard reaming, broaching and filing followed by pulsatile lavage with normal saline (0.9% NaCl) solution and aspiration of any fluid, blood or debris.

Polymethylmethacrylate (Surgical Simplex^® ^P, Stryker International, Limerick, Ireland) was prepared and injected into the medullary canal using a 60 ml syringe. The femoral canal was filled with cement and a cemented modular femoral stem was inserted into the femoral shaft. The femoral anatomic anteversion angle was maintained.

The prosthetic joint was reduced manually. The range of motion and resistance to dislocation were evaluated. A longer or shorter neck was placed if necessary. The joint capsule, muscles, and skin were closed in layers.

In the postoperative period, dogs received tramadol hydrochloride (2 mg/kg BW; Tramal, Searle, São Paulo, Brazil) orally three times a day as needed, meloxicam (0.2 mg/kg BW; Maxican, Ouro Fino, Cravinhos, Brazil) orally once a day for 5 days, and cephalexin (30 mg/kg BW; Cefalexina, EMS, São Paulo, Brazil) orally three times a day for 10 days.

Dogs were housed in individual pens after surgery. Exercise was restricted to two 10-minute leash walks per day during the first postoperative month. After that, dogs had daily unrestricted exercise in the pens.

Limb function was evaluated using a scoring system before surgery and again at 30, 60, 90, 120, 180 and 360 days post-operation. The five-point scoring system used for evaluation at each time point has been designed so that lower scores indicate better outcomes than higher scores (1 = normal, 2 = periodic lameness or gait variance, 3 = moderate lameness or hip joint pain, 4 = severe lameness or reduced weight bearing most of the time, 5 = continuous marked lameness). All the clinical and orthopedic evaluations were performed by the same person.

Pain on palpation was evaluated by a scoring system before surgery and again at 30, 60, 90, 120, 180 and 360 days post-operation. This four-point scoring system was also used for evaluation at each time point and has been designed so that lower scores indicate better outcomes than higher scores (1 = absence of pain, 2 = mild pain, 3 = moderate pain/reaction to manipulation, 4 = severe pain/vocalization/intense reaction to manipulation).

Standard lateral, ventrodorsal extended, and flexed leg radiographic projections of the pelvis were taken before surgery, immediately after surgery, and again at 30, 60, 90, 120, 180 and 360 days post-operation. Additional pelvic radiographs were performed whenever a dog became lame or if existing lameness worsened, did not improve, or dislocation was suspected.

The values for the non-parametric variables, limb function and pain on palpation, were expressed as median and minimum and maximum values. For comparison of the different evaluation moments with the baseline moment, repeated measures analysis of variance (ANOVA) followed by the Student-Newman-Keuls (SNK) test for multiple comparisons at a 5% significance level was used.

## Results

The modular hybrid total hip arthroplasty was performed uniformly in 10 dogs with similar outcomes in seven of them.

They exhibited partial weight bearing in the operated limb on the first postoperative day. Occasional lameness was noted in some of the animals, but it was not observed in any of them after 30 days post-surgery. These dogs had excellent limb function scores from the 60^th ^postoperative day to the last evaluation moment.

Palpation revealed dislocation in two dogs which exhibited severe lameness and pain 2 and 5 days after surgery. Radiographs revealed caudoventral dislocation in one of the dogs and caudodorsal dislocation in the other. Both cases required surgery to reduce the dislocation. Soft tissue imbrication was performed and a longer femoral neck was used in these two dogs. One dog had aseptic loosening and exhibited mild progressive lameness after the 90^th ^postoperative day followed by mild muscle atrophy and radiographic signs of loosening after 120 days of surgery, which led to the decision to convert the THA into a femoral head osteotomy (FHO). Microbiological culture was negative.

The second dog was diagnosed with septic loosening, after 90 days postoperatively. Mild progressive lameness and pain upon joint palpation were observed and muscle atrophy of the proximal thigh was noted. Radiographic examination revealed marked osteolysis around the femoral stem at the bone-cement interface with loss of the femoral calcar bone and periosteal formation. The dog did not present an acceptable recovery despite administration of antibiotics and after 150 days, the THA was converted into an FHO.

Finally, one dog exhibited a typical long oblique femur fracture in the vicinity of the tip of the femoral stem 24 hours after surgery. The dog underwent an exploratory arthrotomy and the implants removed after observation of a cement mantle fracture. The femoral fracture was reduced and stabilized using an 8-hole, 3.5 mm-broad dynamic compression plate with 3.5 mm screws.

**Figure 1:**
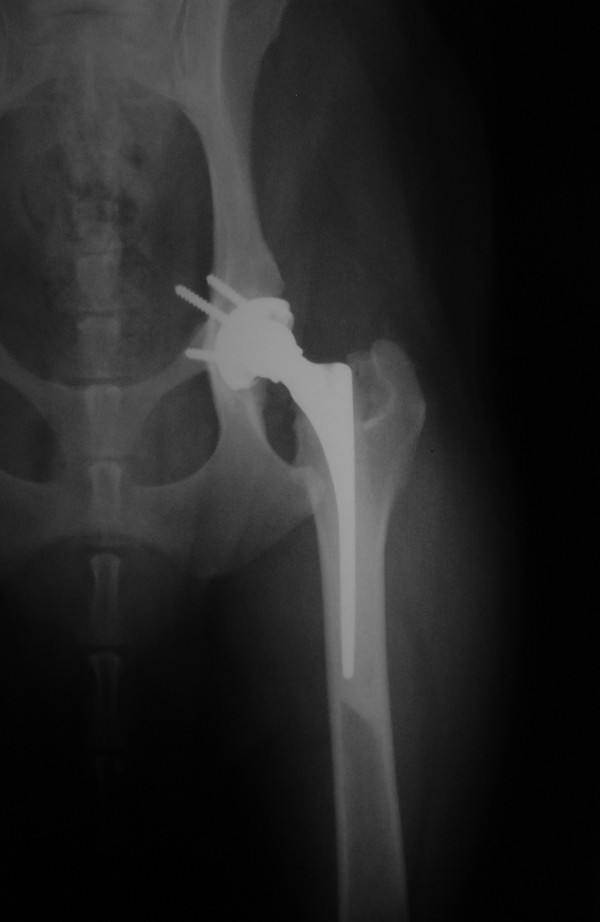
**Ventrodorsal postoperative radiograph after implantation of a modular hybrid total hip prosthesis**. Note the uncemented acetabular cup and the cemented femoral stem.

Radiographic follow-up revealed proper implant orientation and prosthetic joint reduction (Figure 1) in all 10 dogs immediately after surgery. Focal radiographic lucency surrounding the acetabular cup was noted in some dogs, but was non-progressive and no wider than 1 mm except in the two dogs that had implant loosening. No other gross radiographic alterations were observed.

A significant difference (*P *< 0.05) regarding limb function scores was observed between the first evaluation and the subsequent evaluations, which was consistent with an evident improvement in locomotion quality after the 30^th ^postoperative day. No statistical differences were observed between evaluation time points when the first time point was excluded from the analysis; therefore, the scores tended to return to baseline levels along the postoperative evolution.

## Discussion

Total hip arthroplasty is a widely accepted and frequently used surgical technique for the treatment of severe canine hip dysplasia [1,4]. Cemented and cementless systems have been described and clinically used in major veterinary centers of the United States, Australia and Europe [4,9].

In the present work, a modular femoral component was used. This modular system is markedly superior to the fixed-head implants used in previous studies [10]. The modular femoral component reduces the joint laxity and the higher risk of dislocation after THA because it allows the surgeon to choose between different neck lengths even after cementing the definitive stem [13].

Cemented femoral fixation is still the most widely used method in total hip arthroplasty in humans [16]. The literature also contains many reports describing the use of this method in veterinary medicine [2,16,18]. The complication rate associated with cemented femoral fixation is low [14,19]. Conversely, some important complications have been described after the use of uncemented femoral stems [17] including intra-operative femoral fractures upon preparation of the femoral shaft [20] and loosening after adaptive bone remodeling (Stress Shielding) of the proximal portion of the femur [17,20].

Yee et al [12] and Hanson et al [3] have reported that the development of cementless prostheses was based on high rates of aseptic loosening, particularly of the acetabular component.

These aforementioned facts encouraged the development of the present study: here, a cemented femoral component, which is associated with low complication rates, and a cementless acetabular cup were used.

Difficulties in cementing the prosthetic acetabular component have been described [2,8] whereas the use of cementless acetabular components has proven to be simpler and more practical. The use of components from the cementless system results in greater placement precision because of the significantly longer time available to the surgeon when compared with cemented fixation of the acetabular cup. If necessary, the surgeon can replace a cementless acetabular cup more easily than a cemented one [13]. In the present study, we found that cementless acetabulum fixation procedures are simpler than those required for cemented fixation used by our team in a previous work [8].

Lasting and rigid fixation of the acetabular cup without cement has been demonstrated in dogs [3,21]. Different types of prosthetic acetabular cups have been successfully used, such as the press-fit, the threaded, and the screwed models [3,12,22,23]. Superior results have been attained using uncemented acetabular components initially fixed with screws [24] as the initial instability is considered a major cause of loosening of uncemented acetabular components [20]. Therefore, in the present study, an uncemented acetabular component with screw fixation was used.

Hip function is usually restored around eight weeks after total hip arthroplasty [5]. In this study, 7 of 10 animals (70%) showed acceptable hip function with minimal pain and lameness on the first postoperative day and no lameness in the locomotion evaluation at 60 days or eight weeks after the surgery. Normal hip function and no join pain were exhibited in the same 7 dogs after 360 days of surgery; this result is similar to others reported in the literature [3,5,10,11,25].

Few reports describe the use of hybrid prostheses composed of an uncemented acetabular cup and a cemented femoral stem, which complicates the comparison with other studies performed under similar conditions. Previous reports of dogs subjected to implantation of total cementless prostheses have found favorable outcome rates of over 80% [3,4,12,14,22]. However, some major complications specifically related to the procedure of preparation and fixation of the uncemented femoral component have been described [9,26] and reinforces the use of the hybrid prostheses.

Despite the great variety of materials tested as potential bone ingrowth surfaces, porous metal surfaces have proven to be superior [17,21]. Following this trend, the present study used a metallic acetabular component externally coated by a porous fiber titanium mesh. Chen et al. [21] and Heiner et al [27] described the use of a titanium fiber mesh that successfully provided rigid fixation. Bradgon et al [17] described formation of greater amounts and deeper penetration of bone tissue on surfaces coated with titanium fibers when compared to other types of coatings in dogs.

Complications after THA usually range between 5 and 20% [7], although different rates have been described owing to the wide variety of systems used in different populations [2]. In this study, 5 complications were observed in 3 (30%) of the animals that underwent modular hybrid total hip implantation. The complication rate observed can be attributed to inexperience, since our team is currently at the beginning of the learning curve, and to the small number of animals subjected to the prosthetic procedure until now.

Most studies report dislocation as the most common complication after THA, regardless of whether or not the procedure involves cementing [4,13]. In this study, two dislocations were observed. Both dogs exhibited dislocation during the first postoperative week, which coincides with the period of greatest risk of this complication [13]. Improper positioning of prosthetic components is responsible for the majority of the displacements despite the fact that sequelae of previous traumatic events are almost always present [4,13]. Hip laxity due to an excessively short femoral neck is also responsible for higher chances of dislocation in dogs [2,15]. In this study, the two animals that exhibited dislocation were subjected to revision surgery and the positioning of the components was considered appropriate. In spite of that, the femoral neck was lengthened and recurrence of dislocation was not observed after the revision surgery.

Two cases of femoral stem loosening were observed in this study, one septic and one aseptic. Loosening of prosthetic components is considered by some authors the most common complication after THA [5] and is known to be the main long-term complication observed in dogs [18,28]. In this study, the two cases of femoral stem loosening were treated with complete removal of the components, even though recent studies encourage revision surgery [28]. Appropriate positioning of the components and a proper exercise program in the postoperative period reduce the risk of occurrence of this complication. The use of recent modern cementing techniques [1,2,18] and use of uncemented devices [12] are associated with reduced incidence of aseptic loosening.

Femoral fractures after THA represent a major complication, but are rare in dogs [29]. One dog had a femoral fracture after modular hybrid total hip arthroplasty. The most plausible theories regarding the cause of these fractures have been described [29] and the concentration of biomechanical forces in the distal end of the femoral stem is possibly the main reason. However, this does not exclude the possibility of an associated traumatic event. The dog was properly treated with complete removal of the femoral and acetabular components followed by femoral osteosynthesis using bone plate and screws.

Cementless THA has been used increasingly in human medicine for over 10 years especially because it is long-lasting [30]. Similar outcomes have been reported in the veterinary literature [4,14,17]. The present study sought to merge the few complications related to the use of a cemented femoral stem component [14] with the clear advantages of using an uncemented acetabular component [12].

Here, we describe a modular hybrid total hip arthroplasty procedure which is seldomly reported in the veterinary literature and provide several advantages for total hip replacement. The hybrid combination increases the surgeon's options during both primary joint replacement and revision surgeries, emphasizing the importance and necessity of studying hybrid systems.

During this work, the use of a modular hybrid total hip arthroplasty technique in dogs yielded excellent functional outcome of the operated hips. This result encourages the development and dissemination of this technique.

## Competing interests

The authors declare that they have no competing interests.

## Authors' contributions

BWM, CVSB, GJCP, DC, and JJTR were members of the surgical team. BWM was responsible for postoperative care of the patients during the twelve-month follow-up period and was in charge of collection of all data, which included recording the clinical evolution of the dogs and performing the theoretical study that accompanies the process; he was also responsible for bibliographical review and manuscript drafting. CVSB is the group leader and advised the year-long research, suggested bibliography, supervised the process and edited the manuscript; she was responsible for supervision of the manuscript drafting and final approval of all of its contents. GJCP is the human orthopedic surgeon invited to participate in this research and actively collaborated with suggestions and discussions regarding the surgical procedures. DC was the anesthesiologist in charge during the surgical procedures. MJM was responsible for diagnostic imaging during the study. JJTR supervised the process and edited the manuscript. CRP was responsible for the statistical analysis of the present study.

## Authors' information

The manuscript entitled "Modular Hybrid Total Hip Arthroplasty. Experimental Study in Dogs" is the result of the collective effort by the seven authors undermentioned, which fully agree with the final manuscript being submitted on their behalf.

BWM, DVM, MSc, PhD

CVSB, DVM, MSc, PhD

GJCP, MD, MSc, PhD

DC, DVM, MSc

MJM, DVM, MSc, PhD

CRP, BMath, MSc, PhD

JJTR, DVM, MSc, PhD
